# Lessons learned from the development of a new methodology to assess missed opportunities for vaccination in Latin America and the Caribbean

**DOI:** 10.1186/s12914-015-0043-1

**Published:** 2015-02-21

**Authors:** Martha Velandia-González, Silas Pierson Trumbo, José Luis Díaz-Ortega, Pamela Bravo-Alcántara, M Carolina Danovaro-Holliday, Vance Dietz, Cuauhtémoc Ruiz-Matus

**Affiliations:** Comprehensive Family Immunization Unit, Pan American Health Organization/World Health Organization, 525 23rd St, NW, Washington, DC 20037 USA; Vanderbilt School of Medicine, Nashville, TN USA; Center for Research on Infectious Diseases (CISEI in Spanish), National Institute of Public Health (INSP), Mexico City, Mexico; Centers for Disease Control and Prevention, Atlanta, GA USA

**Keywords:** Missed opportunities for vaccination, National immunization programs, Undervaccination, Immunization surveys

## Abstract

The Pan American Health Organization recently developed a practical guide for evaluating missed opportunities for vaccination among children aged <5 years. A missed opportunity occurs when an individual eligible for vaccination has contact with a health facility and does not receive a needed vaccine, despite having no contraindications. In this article, we discuss the strengths and limitations of this new methodology and present lessons learned from recent studies on undervaccination in Latin America. Our findings should be useful to countries embarking on assessing the magnitude and the causes of missed opportunities for vaccination children experience at health facilities.

## Background: the expanded program on immunization in the Americas

The Pan American Health Organization (PAHO) is an international public health agency with more than 110 years of experience working to improve the health of all people in Latin America and the Caribbean (LAC). In 1977, the Directing Council of PAHO, composed of ministers of health of Member States, passed a resolution establishing the Expanded Program on Immunization (EPI) in (LAC) [1,2]. Thirty-six years later, the Region’s accomplishments include the elimination of polio, measles, and rubella and drastic reductions in morbidity and mortality due to vaccine-preventable diseases (VPDs) [1-3]. Immunization programs in LAC are generally sustainable, autonomous, and among the world leaders in introducing new vaccines and passing legislation that protects immunization as a public good [4,5].

**Figure 1:**
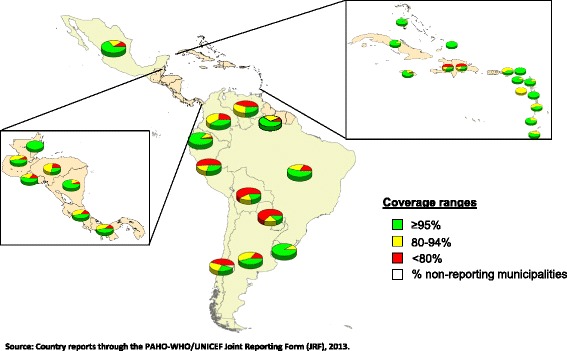
**Municipalities with DTP3 coverage levels in children aged <1 year, LAC, 2012.** Source: Country reports through the PAHO-WHO/UNICEF Joint Reporting Form (JRF), 2013.

One challenge that remains in LAC is ensuring that all children have equal access to immunization services (Figure 1). In 2012, 50% of the 14,716 municipalities in LAC reported DPT3 coverage <95% and 23% reported coverage <80%. Municipalities with <95% DPT3 coverage contain approximately 61% of children in LAC aged <1 year, while 20% of children live in municipalities with <80% [6]. These underperforming municipalities are at risk for the resurgence of VPDs that have been eliminated, eradicated, or are under epidemiological control.

Determinants of immunization coverage are complex and may be interrelated. A recent review of 202 studies identified 838 factors associated with undervaccination [7]. Broadly, the reasons for undervaccination can be grouped into factors related to health workers and supply and those related to users and demand. Recent studies in Colombia, El Salvador, and Guatemala found that most of the population considered vaccines important and effective, but identified barriers in both the supply of and demand for services resulting in many children failing to be fully vaccinated [8-10]. The range of immunization barriers within and among countries underscores the need for local-level studies and tailored interventions to improve delivery of routine immunization services [7].

## Missed opportunities for vaccination

Strategic Objective 3 of the *Global Vaccine Action Plan (GVAP)* calls for the benefits of immunization to be distributed equitably to all people [11]. PAHO, in its *Regional Immunization Vision and Strategy,* shares this goal [12]. PAHO and other partners have helped LAC countries to implement plans of action to raise immunization coverage in vulnerable municipalities. Countries are encouraged to determine local causes of undervaccination and to implement interventions to overcome barriers to achieving high vaccination coverage.

A missed opportunity for vaccination (MOV) occurs when a person who is eligible for vaccination has contact with a health facility and is not vaccinated, despite not having any contraindications to receive that vaccine dose [13,14]. Throughout the 1980s and 90s, operational studies in LAC identified MOVs as an important reason for undervaccination [13-26]. The results of those early MOV evaluations were rarely published in journals but were captured in summaries compiled by PAHO and in technical documents used by the EPI, and demonstrated high MOV rates, ranging from 34-77% in 10 countries [15]. The primary cause of MOVs was the application of false contraindications by health care workers in four countries (El Salvador, Guatemala, Honduras, and Peru), staff attitudes inhibiting immunization in four countries (Bolivia, Colombia, Ecuador, and Venezuela), and logistical issues in two countries (Mexico and Nicaragua). Consumer-related causes, including not prioritizing vaccination, or not believing that vaccination is necessary (0-14%), were proportionally lower in all countries [19]. In response to these data, many LAC countries implemented interventions that reduced MOVs and increased coverage. In El Salvador, evaluations conducted after implementing a number of interventions aimed at stimulating demand and improving delivery showed a reduction in MOVs from 45% to 14% among children aged <5 years [15]. Similarly, Peru reduced MOVs in women of childbearing age and children aged <2 years from 52% in 1990 to 13% in 1995 following the implementation of corrective strategies [26].

In response to recent country requests for assistance in conducting MOV studies with the goal of increasing immunization coverage in vulnerable municipalities, PAHO is making available a standardized methodology for evaluating MOVs in children aged <5 years in primary and secondary health facilities and for evaluating the vaccine-related attitudes and knowledge of health workers [27]. The methodology was adapted from the original WHO methodology published in 1988 and other immunization studies implemented in the Region and takes into account best practices in immunization surveys from LAC [13]. The methodology was first piloted in the Dominican Republic in October 2012 (Garib Z, et. al., “Missed opportunities for vaccination in the Dominican Republic: results of an operational investigation”, in preparation).

In this paper, we describe the updated methodology and questionnaires for evaluating MOVs, including the process of developing these tools; share lessons learned from the pilot project and other recent immunization studies conducted in LAC; and discuss the limitations and advantages of the updated methodology. This analysis should be useful to countries in LAC and in other regions that seek to determine why eligible children are not vaccinated despite having contact with health facilities.

## Development and testing of the MOV tool

PAHO’s updated methodology for identifying MOVs was developed from May 2012 to June 2013. To identify best practices and major lessons from previous MOV studies, we reviewed articles on undervaccination and MOVs in the regional literature from 1980–2013. Using Medline, PubMed, Google Scholar, Artemisa, and other search engines, we identified 117 articles and studies concerning MOVs in children aged <5 years (Table 1).

**Table 1:** **Missed opportunities for vaccination and factors associated with undervaccination in children aged <5 years: Literature search--Americas Region, 1980–2013** ^**(a)**^

**Results obtained**	**Medline**	**Other search engines** ^**(b)**^	**Grey literature** ^**(c)**^	**Total**
Total results	1084	13200	30	14314
“Suggestive” title ^(d)^	178	1597	25	1800
Not related ^(e)^	90	1295	0	1385
Duplicates	9	88	0	97
Not found	30	146	0	176
Not vaccines of the national immunization program	7	18	0	25
Included in the search	42	50	25	117

^(a)^ Descriptors used: missed opportunities for vaccination; vaccination knowledge, attitudes, and practices; vaccination coverage, causes of no vaccination; children younger than 5 years. Search limits: period 1980–2013; studies written or published in Spanish, English, Portuguese, or French.

^(b)^ Artemisa, Lilacs, Bireme, Google Scholar, Redalyc.

^(c)^ The term “grey literature” includes references found in bibliographies of published articles, technical documents and presentations available on Google Scholar, and documents available on PAHO and country websites.

^(d)^ Articles with a “suggestive title” were considered those that might reasonably have been considered to concern missed opportunities for vaccination.

^(e)^ Articles in the “not related” category employed some of the descriptors mentioned. However, these articles were excluded for one or more of the following reasons: the country studied was out of the Americas Region; the age group studied was not children aged <5 years; and/or the focus of the article was not on factors associated with MOVs or undervaccination.

Based on our review of available data, we developed the study methodology and two questionnaires: one to measure MOVs in children aged <5 years and women of childbearing age and one to evaluate the knowledge, practices, and attitudes of health workers. A guiding principle for the inclusion of information to be collected was its usefulness in the field and its potential for identifying corrective measures. The method was designed such that both questionnaires would be implemented on the same day at the same health facility, with the first being administered by interviewers to caregivers of children aged <5 years and the second being anonymously completed by individual health workers. The methodology seeks information from a broad range of participants and is designed to evaluate health practices in visits intended for vaccination and in those sought for other reasons (e.g., well child check-ups). Caregivers of children aged <5 years are eligible to participate following a visit to a health center for any reason. Healthcare professionals who do not routinely administer vaccines, including those who work in nutrition and well child clinics, may also be included in the health worker surveys.

The tool consists of case definitions, methods, brief questionnaires for assessing MOVs, and guidelines for analyzing and presenting findings. The methodology and questionnaires were originally written in Spanish and later translated into English. Eighteen immunization professionals from Latin American countries, PAHO, WHO, and CDC reviewed the tools to ensure clarity and technical accuracy, and approximately 40 supervisors, interviewers, and analysts from a professional polling company in the Dominican Republic reviewed the questionnaires.

With approval from the ethics committee of Mexico’s National Institute of Public Health, the questionnaires were tested in Morelos, Mexico in August 2012. Among 150 eligible participants contacted in four health facilities, 129 (86%) agreed to participate, with a higher rate of participation in rural than in urban areas. Seventy-three (78%) of 94 health workers completed the questionnaire. This pilot project enabled the further refinement of the questionnaires and the methodology.

In October 2012, the Dominican Republic piloted the updated methodology using the methodology and questionnaires written in Spanish. In 99 health centers in low-coverage municipalities, 1500 parents and guardians of a child aged <5 years were interviewed and 398 healthcare professionals completed the health worker survey. Of 782 opportunities for 527 eligible children to receive needed vaccines, a total of 262 MOVs was observed (Garib Z, et. al., “Missed opportunities for vaccination in the Dominican Republic: results of an operational investigation”, in preparation). To evaluate the completeness, implementation and understanding of the methodology, PAHO professionals participated in all stages of the evaluation. Implementation was considered successful: the assessment was feasible to implement in two weeks, target sample sizes were obtained, and a large proportion of health workers participated, recognized the findings as problems in their health facilities, and proposed solutions to these problems. Additionally, results were useful to decision-makers at the sub-national and national levels. However, modifications to the surveying instruments had to be made. Most significantly, questions to measure MOVs in women of childbearing age were eliminated due to the low proportion of these women (6%, n = 81/1387) bringing vaccination cards to health facilities (Garib Z, et. al., “Missed opportunities for vaccination in the Dominican Republic: results of an operational investigation”, in preparation).

## Description of methodology, survey tools, and guidelines to the countries

The methodology allows for a cross-sectional evaluation of MOVs. Because the evaluation serves as an operational tool for the identification of MOVs in municipalities that do not meet target coverage levels, quota sampling rather than probability sampling is recommended. Geographical areas (municipalities) are first selected based on coverage rates, indices of unmet basic needs, and other indicators. Health facilities are then selected, taking into account the proportion of the population residing in rural versus urban areas and the proportion of patients who use hospitals versus primary care centers.

**Figure 2:**
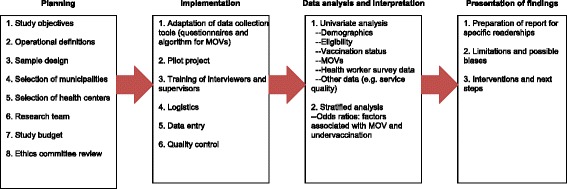
**Phases for the evaluation of missed opportunities of vaccination, principal components of the PAHO methodology.**

The methodology is divided into four main sections that describe how to plan, implement, analyze, and present the study’s findings (Figure 2). Because studies on MOVs and immunization require significant investments in financial and human resources, the survey tools were designed to assess multiple aspects of programs. Unlike the WHO’s previous methodology, the updated methodology includes questions on a range of immunization program attributes of interest to countries (e.g., service quality, impact of communication strategies); a detailed explanation of the sampling procedure; a discussion of ethical considerations; an Excel tool with defined algorithms to facilitate data analysis; and a list of valid and false contraindications to vaccination [28]. The updated methodology also allows for the evaluation of attitudes, knowledge, and practices of health workers, thereby facilitating the design of specific training and supervision activities for healthcare professionals. Finally, the methodology includes indicators for evaluating other aspects of the EPI, including service quality, health worker practices, and parental attitudes and practices related to immunization.

**Figure 3:**
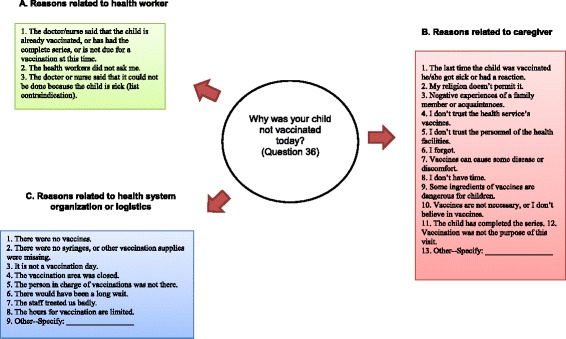
**Reasons for missed opportunities for vaccination in health exit survey by category.**

The questionnaire contains 52 questions pertaining to sociodemographic data, parental immunization attitudes and practices, perceptions of service quality, and the evaluation of MOVs, as well as a space for interviewers to transcribe dates of vaccination from the child’s vaccination card. MOVs are assessed using a simple algorithm. The participant is asked if his or her child was vaccinated today and, if not, why vaccination did not occur. Interviewers code responses into categories of reasons related to health workers, caregivers, or the health system (Figure 3). Based on the child’s immunization history, it can then be determined if a MOV occurred. Each child can have multiple MOVs if more than one indicated vaccine was not administered.

The health worker questionnaire measures health worker knowledge, attitudes, and practices pertaining to immunization, with additional questions for vaccinators. According to the methodology, health workers are considered to possess attitude, knowledge, or decision-making barriers to vaccination if they incorrectly answer >20% of questions in these sections.

To implement the assessment, a country must adapt the questionnaires and MOV algorithm to its vaccination schedule. Definitions for a timely dose, eligible child, and window of opportunity for a timely dose must all be established. The methodology provides guidelines to aid investigators in determining eligibility, timely doses, and windows of opportunity. The country should then select an implementation team. Implementation teams should consist of a general coordinator, supervisors, interviewers, and data entry personnel (if data are collected using paper forms), and the inclusion of a statistician in the study team is recommended. The team may be composed of non-immunization health professionals, or the country may hire an independent polling company or an academic institution to conduct the assessment. Training sessions for team members, a pilot test, and procedures to ensure data quality are required. Before implementing the study, investigators must ensure that it will be conducted according to national regulations for the use of health data. Investigators are encouraged to conduct univariate and stratified analyses to identify factors associated with MOVs and undervaccination in the surveyed population, with the understanding that the results are not generalizable to the entire country as sampling is non-probabilistic. The final step is the preparation of a report that facilitates the design of specific strategies to reduce MOVs. This report should be presented to subnational and national immunization program managers, other health authorities, and partners where applicable.

## Lessons learned

Foremost among the many lessons learned while developing this MOV guide and implementing it in the Dominican Republic, as well as from recent studies on undervaccination in LAC (Table 2), is that the EPI and the Ministry of Health must lead the assessment, even if they are not the main implementers. EPI involvement is critical in adapting the survey instruments to local realities, developing and implementing interventions to raise coverage rates, and monitoring their usefulness, as well as for satisfying ethical requirements. In addition, to avoid duplication of effort, care must be taken to coordinate, engage, and communicate the evaluation plan with other country-level immunization partners.

**Table 2:** **Lessons learned from study on missed opportunities of vaccination in Dominican Republic (October 2012) and from other immunization surveys in Latin America (2010–2011)**

**Political support**	-EPI should lead the study, even when international technical or financial support is available.
	-Cooperation among governmental agencies is required, particularly ministries of health, finance, and statistics.
	-EPI should start ethics approval process as soon as possible to prevent delays.
**Country adaptation of instruments**	-Sampling procedure must be carefully determined, preferably with assistance from a government statistician.
	-Surveys must be adapted to take into account differences in culture, local language, and EPI schedules.
	-Algorithms/syntaxes for determining missed opportunities must take into account replacement vaccines. A professional with expertise in computers and statistical programs should participate in the data analysis and address problems as they arise.
	-Survey questions must be understandable to all people regardless of education level.
	-Avoid technical language.
	-Professionals in different disciplines, including those outside of healthcare, should review questionnaires.
	-Pilot project or focal groups conducted prior to study implementation are key to adapting surveying instruments to local realities.
**Implementation**	-Role-play and the use of sample vaccination cards are useful tools in training interviewers to properly conduct interviews.
	-Explaining the study’s potential impact to interviewers promotes their interest in and commitment to the study.
	-Request feedback on surveying tools from interviewers/supervisors (many are parents and provide useful feedback).
	-EPI should notify health facilities that they have been selected for a survey 1–2 days before implementation.
	-New technologies (e.g. Palm Pilots) may reduce paperwork and data entry errors and enable local decision makers to access to data more quickly.
	-A polling company, if cost-effective, allows EPI professionals to focus on technical issues and provides an independent perspective and surveying expertise.
	-Properly-trained supervisors are key to ensuring that the questionnaire is properly administered and to minimizing data-entry errors.
	-Supervisors should seek to have health facility directors distribute the health worker survey at grand rounds or during shift changes.
**Data analysis and design of Interventions**	-EPI professionals at all levels--local, sub-national, national--should review results and be asked for suggestions to reduce MOVs.
	-To promote their involvement in the study and the design of local interventions, directors of evaluated health facilities should receive results.
	-Results should be published to increase understanding on the causes of under vaccination, establish a baseline for progress, and advocate for funding.
	-Reports should not be overly detailed. Too much information may obscure the study’s principal findings.
	-Report should advocate that study be repeated once interventions are implemented so that progress can be measured.

It is important that questionnaires avoid technical language and reflect local language and circumstances and that representatives from local communities be involved in the process of reviewing the questionnaire. The Dominican Republic benefited greatly from having the questionnaires reviewed by more than 40 individuals, including EPI officials, nurses, and physicians as well as by interviewers, supervisors, and data analysts from the polling company that conducted the evaluation. Similarly, pilot tests are critical to anticipating and correcting problems in the field. In some situations, it may be beneficial to convene focus groups of parents of undervaccinated children to help inform questionnaire design. During a recent study in Colombia, focus group discussions complemented the quantitative data analysis and helped researchers design the survey and formulate hypotheses on barriers to immunization [8].

Countries should consider the advantages and disadvantages of hiring a professional polling company or an academic institution. Polling companies provide an independent perspective and expertise in surveying that allows the EPI to concentrate on technical issues. National authorities and international donors may also have more confidence in an assessment conducted by an external evaluator. However, studies conducted by polling companies or academic institutions are usually more expensive and may require more training in EPI issues. For this reason, countries with universities with postgraduate programs in epidemiology or with existing knowledge or research interests in the EPI may be viable options to implement the study. Regardless of the option chosen, the importance of properly trained supervisors and interviewers cannot be overemphasized. Interviewers and supervisors should practice administering the questionnaire to each other and transcribing sample vaccination cards, preferably ones with errors and complications (e.g., substitution vaccines or crossed-out entries).

In the Dominican Republic, supervisors established good relationships with health center directors, provided constructive feedback to interviewers, and carefully validated all data on the assessment day to minimize errors. To maximize the number of interviews conducted, supervisors strategically positioned interviewers at the locations in the facility where children aged <5 years were most likely to be encountered (e.g., outside the pediatric department or vaccination post). A final consideration on implementation is the use of electronic technologies (tablets, smart phones, etc.) for data collection. In Guatemala, investigators used handheld Palm Pilot Personal Digital Assistant (PDA) devices to collect data, thereby reducing paperwork and allowing results to be delivered promptly to national authorities [9].

Lessons on data analysis include the importance of having tools to determine precisely what constitutes a MOV. Immunization schedules in LAC include as many as 13 antigens, and it can be challenging to determine whether a particular child is fully vaccinated for his or her age. To address this problem, PAHO’s Excel tool for assessing MOVs must be carefully aligned to the adapted country questionnaire and database before starting the evaluation. A professional with expertise in databases should be available to address potential problems.

Implementers should prepare a brief report highlighting major findings for national health authorities and a more detailed report for the EPI and local program managers. In the Dominican Republic, results were presented to both national and subnational EPI managers. During our pilot testing, subnational officials, many of whom are responsible for immunization services in evaluated health centers, suggested interventions and helped ascertain underlying factors related to identified barriers. Moreover, the inclusion of local-level immunization officials in the MOV assessment increases the involvement and commitment of the officials who are ultimately responsible for implementing interventions.

Lastly, countries should document studies they conduct on MOVs and undervaccination. The limited number of published studies in developing countries, particularly in LAC, that evaluate immunization programs, validate coverage data, or assess the effectiveness of interventions is well known [29,30]. Among other benefits, increased documentation of operational studies on immunization will help countries establish a baseline for progress, advocate for increased political commitment and external funding, promote evidence-based decision-making, and share experiences with the rest of the immunization community.

## Advances, limitations, and next steps

This methodology should enable countries to quickly identify and correct immunization barriers that result in MOVs. It is also a valuable tool for countries wishing to operationalize the *GVAP,* which calls for a better understanding of the causes of undervaccination and for equitable access to immunization services.

The MOV methodology developed by PAHO is standardized, adaptable, and designed as an operational research tool. By simultaneously evaluating MOVs and the knowledge, practices, and attitudes of health workers and caregivers, the new methodology facilitates the design of more effective training and communication activities for health professionals [COMMVAC] [31].

The evaluation in the Dominican Republic exemplifies how the tool may be used to identify cost-effective interventions to include in EPI national plans of action. For example, results from the pilot project in the Dominican Republic show that the majority of MOVs occurred because health workers neglected to ask caregivers for the child’s vaccination card or did not review it properly. Therefore, one cost-effective remedy for increasing coverage in vulnerable areas in the Dominican Republic may be the increased sensitivity of health workers to the importance of requesting and carefully reviewing the vaccination card at each health care encounter. Further research is required to determine if this cause of undervaccination is also a major cause of undervaccinated children elsewhere in LAC.

There are four principal limitations associated with the MOV methodology. First, the methodology is not intended to evaluate populations without access to health services. While participants are asked how far they traveled to seek services, the study is conducted in health facilities and therefore precludes evaluation of populations who do not have access to those facilities. Consequently, a door-to-door approach may be more appropriate in settings where limited access is suspected to be a major barrier. Second, the sample is only representative of children aged <5 years who have contact with non-randomly selected health facilities at the time of the evaluation. As a cross-sectional study tool, the methodology is useful for determining associations among MOVs and the reasons that vaccination did not take place at that visit. However, causal associations between these reasons and the vaccination status of children, and between MOVs and demographic data, are not possible. Third, although the updated methodology does not require the direct observation of health care workers, health workers will know that a study is taking place at their center. Consequently, they may act more vigilantly, leading to an underestimation of MOVs for reasons related to health professionals. Finally, as with other health surveys, selection and recall biases may be present [32].

Despite these limitations, the updated MOV methodology has generated considerable interest among LAC countries wishing to attain more equitable immunization coverage rates. The EPI in Guatemala conducted a MOV assessment in 2013, and Panama, Peru, and Bogotá (Colombia) conducted assessments in early 2014. While causes of undervaccination will vary within and among countries, commonalities will also exist. For this reason, PAHO, in conjunction with WHO and CDC, will continue to review studies to generate knowledge about the regional causes of undervaccination. PAHO is also working to make available information on best practices for reducing MOVs and increasing coverage rates, describing how successful interventions are developed, cost-effectively implemented, monitored, and evaluated. PAHO encourages countries to document interventions and to repeat this type of study, ideally with a costing component, in three to five years to evaluate whether the interventions implemented were successful in reducing MOVs and contributed to more equitable immunization coverage rates.

## Abbreviations

PAHO: Pan American Health Organization
LAC: Latin America and the Caribbean
EPI: Expanded Program on Immunization
VPDs: Vaccine-preventable diseases
GVAP: Global vaccine action plan
CIDA: Canadian International Development Agency
CDC: United States Centers for Disease Control and Prevention
MOV: Missed opportunity for vaccination
WHO: World Health Organization

## References

[1] Tambini G, Andrus JK, Fitzsimmons J, Periago M (2006). Regional immunization programs a model for strengthening cooperation among nations. Rev Panam Salud Publica.

[2] Andrus JK, Fitzsimmons J, De Quadros CA, Andrus JK, de Quadros CA (2006). Introduction of new and underutilized vaccines: perspectives from the Americas. Recent Advances in Immunization.

[3] World Health Organization / UNICEF. Global and regional immunization profile: Region of the Americas (2012). http://www.who.int/immunization_monitoring/data/data_regions/en/

[4] Trumbo SP, Janusz CB, Jauregui B, McQuestion M, Felix G, Ruiz-Matus C (2013). Vaccination legislation in Latin America and the Caribbean. J Public Health Policy.

[5] de Oliveira LH, Danovaro-Holliday MC, Sanwogou NJ, Ruiz-Matus C, Tambini G, Andrus JK (2011). Progress in the introduction of the rotavirus vaccine in Latin America and the Caribbean: four years of accumulated experience. Pediatr Infect Dis J.

[6] Pan American Health Organization / World Health Organization. Country reports through the PAHO-WHO/UNICEF Joint Reporting Form (JRF), 2012. http://www.paho.org/hq/index.php?option=com_content&view=article%20&id=2043&Itemid=2032&lang=en.

[7] Rainey JJ, Watkins M, Ryman TK, Sandhu TK, Bo A, Banerjee K (2011). Reasons related to non-vaccination and undervaccination of children in low and middle income countries: findings from a systematic review of the published literature, 1999–2009. Vaccine.

[8] García DAL, Velandia-González M, Trumbo SP, Pedreira MC, Bravo-Alcántara P, Danovaro-Holliday MC (2014). Barriers to immunization and the design of research-based communication strategies in Colombia. BMC Public Health.

[9] Barrera L, Trumbo SP, Bravo-Alcántara P, Velandia-González M, Danovaro-Holliday MC (2014). From the parents’ perspective: a user-satisfaction survey of immunization services in Guatemala. BMC Public Health.

[10] Suárez-Castaneda E, Pezzoli L, Elas M, Baltrons R, Crespin-Elías EO, Rivera Pleitez OA (2014). Routine childhood vaccination program coverage, El Salvador, 2011: in search of timeliness. Vaccine.

[11] World Health Organization. Global Vaccine Action Plan, 2011–2020. http://www.dovcollaboration.org/action-plan/.

[12] Pan American Health Organization. Regional Immunization Vision and Strategy. http://www.who.int/immunization/sage/PAHO_RIVS.pdf.

[13] Sato P. Methodology for the assessment of missed opportunities for immunization. http://whqlibdoc.who.int/hq/1988/WHO_EPI_GEN_88.6.pdf.

[14] Hutchins SS, Jansen HAFM, Robertson SE, Evans P, Kim-Farley RJ (1993). Studies for missed opportunities of immunization in developing and industrialized countries. Bull World Health Org.

[15] The Pan American Health Organization/ World Health Organization (1991). Missed opportunities for vaccination in the Americas: diagnosis and interventions, 1988–1990. EPI Newsl.

[16] Zessig O, Hoysler R, Da Cunha C, Castillo-Solorzano CJ, Olive JM (1990). Missed opportunities for vaccination in Guatemala. EPI Newsl.

[17] Pan American Health Organization and Dirección Nacional de enfermedades transmisibles del Ministerio de Salud de Nicaragua. Oportunidades perdidas de vacunación en niños que acuden a centros y puestos de salud en áreas de las regiones I, II, III, IV, V y VI. EPI/TAG/88/04.

[18] Meneses Reyes CD, Díaz Ortega JL (1996). Metodología e instructivo para encuestas de oportunidades perdidas de vacunación.

[19] Rodríguez GMA (2001). Magnitud y causas de oportunidades perdidas en vacunación en población menor de dos años en América. CES Med.

[20] The Pan American Health Organization/ World Health Organization (1995). Nicaragua: strategies to reduce missed opportunities to vaccinate. EPI Newsl.

[21] Diaz-Ortega JL, Camacho AML, Muñoz BS, Santis W. Oportunidades perdidas de vacunación en menores de cinco años en la Ciudad de México. Consejo Nacional de Vacunación, 1991.

[22] Moguel-Parra G, Martínez G, Santos-Preciado JI (1992). Factores que influyen en la inmunización de los niños en la consulta externa de un hospital pediátrico. Bol Med Hosp Infant Mex.

[23] López-Ortíz A, López-Andrade MG, López-Torres J, Díaz-Ortega JL (1992). Oportunidades perdidas de vacunación. Gaceta Vacunación.

[24] Avila-Figueroa C, Navarrete-Navarro S, Ramírez-Galván L, Baltazar-López A, López-Serrano M, Santos-Preciado JI (1992). Inmunizaciones en niños hospitalizados y de consulta externa: reducción de las oportunidades perdidas de vacunación. Bol Med Hosp Infant Mex.

[25] The Pan American Health Organization/ World Health Organization (1990). Study of missed vaccination opportunities in Colombia. EPI Newsl.

[26] The Pan American Health Organization/ World Health Organization (1996). Missed opportunities to vaccinate in Peru. EPI Newsl.

[27] Pan American Health Organization. Methodology for the evaluation of missed opportunities for vaccination. http://www.paho.org/hq./index.php?option=com_docman&task=doc_download&gid=23943&Itemid=270&lang=es.

[28] Centers for Disease Control and Prevention. Chart of contraindications and precautions to commonly used vaccines. http://www.cdc.gov/vaccines/recs/vac-admin/contraindications-vacc.htm.

[29] Miles M, Ryman TK, Dietz V, Zell E, Luman ET (2013). Validity of vaccination cards and parental recall to estimate vaccination coverage: a systematic review of the literature. Vaccine.

[30] Ryman TK, Dietz V, Cairns KL (2008). Too little but not too late: results of a literature review to improve routine immunization programs in developing countries. BMC Health Serv Res.

[31] Willis N, Hill S, Kauffman J, Lewin S, Kis-Rigo J, De Castro Freire SB (2013). “Communicate to vaccinate:” the development of a taxonomy of communication interventions to improve routine childhood vaccination. BMC Int Health Hum Rights.

[32] Cutts FT, Izurieta HS, Rhoda DA (2013). Measuring coverage in MNCH: design, implementation, and interpretation challenges associated with tracking vaccination coverage using household surveys. PLoS Med.

